# Medical recommender systems based on continuous-valued logic and multi-criteria decision operators, using interpretable neural networks

**DOI:** 10.1186/s12911-021-01553-3

**Published:** 2021-06-11

**Authors:** Juan G. Diaz Ochoa, Orsolya Csiszár, Thomas Schimper

**Affiliations:** 1 PerMediQ GmbH, Salzbergweg 18, 85368 Wang, Germany; 2 Faculty of Basic Sciences, University of Applied Sciences, Esslingen, Esslingen, Germany; 3 Physiological Controls Research Center, Óbuda University, Budapest, Hungary; 4 Knowledgepark GmbH, Leonrodstr. 68, 80636 Munich, Germany

**Keywords:** Recommender system, Artificial intelligence, Deep learning, Continuous valued logics, Data privacy, Health records

## Abstract

**Background:**

Out of the pressure of Digital Transformation, the major industrial domains are using advanced and efficient digital technologies to implement processes that are applied on a daily basis. Unfortunately, this still does not happen in the same way in the medical domain. For this reason, doctors usually do not have the time or knowledge to evaluate all alternative treatment options for each patient accurately and individually. However, physicians can reduce their workload by using recommender systems, still having every decision under control. In this way, they also get an insight into how other physicians make treatment decisions in each situation. In this work, we report the development of a novel recommender system that uses predicted outcomes based on continuous-valued logic and multi-criteria decision operators. The advantage of this methodology is that it is transparent, since the model outcomes emulate logical decision processes based on the hierarchy of relevant physiological parameters, and second, it is safer against adversarial attacks than conventional deep learning methods since it drastically reduces the number of trainable parameters.

**Methods:**

We test our methodology in a patient population with diabetes and heart insufficiency that becomes a therapy (beta-blockers, ACE or Aspirin). The original database (Pakistan database) is publicly available and accessible via the internet. However, to explore methods to protect the patient's identity and guarantee data privacy we implemented a methodology on a variable-by-variable basis by fitting a sequence of regression models and drawing synthetic values from the corresponding predictive distributions using linear regressions and norm rank. Furthermore, we implemented a deep-learning model based *on* logical gates modeled by perceptrons with fixed weights and biases. While a first trainable layer automatically recognizes a meaningful parameter hierarchy, the implemented Logic-Operator Neuronal Network (LONN) simulates cognitive processes like a rational, logical thinking process, considering that this logic is joined by fuzziness, i.e., logical operations are not exact but essentially fuzzy due to the implemented continuous-valued operators. The predicted outcomes of the model (kind of therapy-ACE, Aspirin or beta-blocker- and expected therapy time of the patient) are then implemented in a recommender system that compares two different models: model 1 trained on a population excluding negative outcomes (patient group 1, with no patient dead and long therapy times) and a model 2 trained on the whole patient population (patient group 2). In this way, we provide a recommendation of the best possible therapy based on the outcome of the model and the confidence of this recommendation when the outcome of model 1 is compared with the outcome of model 2.

**Results:**

With the applied method for data synthetization, we obtained an error of about 1% for all the relevant parameters. Furthermore, we demonstrate that the LONN models reach an accuracy of about 75%. After comparing the LONN models against conventional deep-learning models we observe that our implemented models are less accurate (accuracy loss of about 8%). However, the loss of accuracy is compensated by the fact that LONN models are transparent and safe because the freezing of training parameters makes them less prone to adversarial attacks. Finally, we predict the best therapy as well as the expected therapy time. We were able to predict individualized therapies, which were classified as optimal (binary value) when the prediction fully matched predictions made with models 1 and 2. The results provided by the recommender system are displayed using a graphical interface. The current is a proof of concept to improve the quality of the disease management, while the methods are continuously visualized to preserve transparency for the customers.

**Conclusions:**

This work contributes to simplify administrative functions and boost the quality of management of patients improving the quality of healthcare with models that are both transparent and safe. Our methodology can be extended to different clinical scenarios where recommender systems can be applied. The acceptance and further development of the app is one of the next important steps and still requires further development depending on specific requirements of the health management, the physicians or health professionals, and the patent population.

**Supplementary Information:**

The online version contains supplementary material available at 10.1186/s12911-021-01553-3.

## Background

The management and quality of hospital services depend to a large extent on individual medical decisions. For example, based on their experience, each physician may select treatments coded into specific keys (Treatment Keys, or TK) for disease management and the cost of treatment; each of these treatments is then combined with a cost refund respectively which is encoded in specified keys in the Electronic Health Records (EHR) and on medical bills. These keys would have an ‘economic effect’ on the health system [1]. The reimbursement system's behavior, in some cases, enables what physicians could do to offer more health services to help a patient, regardless of whether the additional care is economically optimal. At the same time, physicians can also ignore their colleagues' experiences by using alternative treatments that might be more suitable for treating a disease. Other deficiencies may stem from the fact that physicians are either overloaded by a large number of patients and/or by several therapeutic options to consider, which is part of a portfolio that has more than 100 different alternative treatments per patient [2]. Because of this, physicians do not usually have the time or knowledge to evaluate all of the different alternative options accurately and individually.

By using recommender systems, physicians can reduce their workload, yet still have each decision made under their control. They will also get an insight into how other physicians recommend a treatment in the same given situation.

Nowadays, recommender systems have proven to be invaluable for online users to cope with information overload in many different fields (e.g. e-commerce, decision support systems, etc.) [3, 4]*.* However, the architecture of recommender systems and their evaluation in real-world problems is still an active area of research. We have investigated and implemented a recommender system intending to transfer this technology in the field of medicine to support physicians seeking to predict the *rating* or *preference* of a treatment key for new patients.

Recommender systems in medicine are not new. There are about 911 search items in published medical journals, with articles reporting recommender systems for patients using personal health systems [5]. Until the last few years, most of these techniques were used for analyzing rich EHR-data based on traditional machine learning and statistical techniques such as logistic regression, support vector machines (SVM) [6], and random forests. However, recently, deep learning techniques have achieved great success in many domains through deep hierarchical feature construction and capturing long-range dependencies in data effectively, particularly for the analysis of EHRs in medicine [7]. For a recent review article on recommender systems in healthcare see e.g. Tran et al*.* [8].

Here, we are introducing a new type of recommender system which is a combination of methods to generate synthetic populations while keeping personal data protected when needed for Artificial Intelligence (AI) applications and the use of novel methods based on continuous-valued logic and multi-criteria decision operators aimed for robust, safer, and more understandable use of deep learning. By combining neural networks with continuous logic and multi-criteria decision-making tools, thus reducing in this way the black-box nature of neural network models [9–12]. By doing this, we are exploring the (often overlooked) possibility of combining neural networks with continuous logical systems. This strategy provides a clear advantage in the medical field since it is a system that, due to its nature, can be easily understood by physicians and/or medical practitioners, who often make their decisions relying on continuous logical rules. We aim to reach more transparency of AI applications in medicine while preserving efficient deep learning methods. The synthetic populations are mainly used for training the Deep Learning machinery. They are completely anonymized yet keep their original structure of the original data sample used for ML use.

Our customers consist of physicians handling individual patient diagnoses. In this way, by providing these recommendations, we are seeking to reach both an economic and clinical efficacy:

- Persuade physicians to make use of best-suited treatment keys aims to reduce the costs of the patient management.
- Improve the management of a diagnosis of the disease by helping the physicians to discover additional keys that could improve the treatment of patients.

These two items seem to be contradicting since one aspect has an economic motivation while the second aspect has a medical nature. But they represent the typical conflict of interest that each physician faces daily. Physicians should act accordingly to the following principles:

- Best medical care for the patient with optimum economic impact (i.e. cost efficiency) for those who have to pay the bill finally. So, if there are two equal treatments available, the system should recommend the cheaper one with the same if not better treatment quality result than the more expensive option.
- Depending on the health system of a particular country and how billing/charging is carried out, there may be another conflict that the physician has to contend with: optimizing the cost-income ratio of the organization that is treating patients.

As the system is learning from the best physicians in the community, the recommendations made could be seen as the best advice from the best physicians around.

Our recommender system consists of a collaborative filtering approach. The goal is to build a model from the past behavior of several physicians selecting treatment keys that are correlated to the patient’s diagnosis keys (codified by the ICD system). Therefore, when a new patient with specific ICDs enters the system, the physician is shown a recommendation list of the plausible treatment keys for this patient with the most effective therapy. Thus, to extract structured medical concepts, such as diseases and treatment procedures, we use single-concept extraction in electronic medical records [6].

In the next section, we will introduce the architecture implemented in this project. After that, we will describe the implemented methodology and present the validation results compared to conventional dense networks. Then we will further show the visualization method implemented in a recommender system. Finally, we will discuss the implication of this implementation and conclude with a deviation on how this methodology can be further improved.

## Methods

### Solution architecture

The implemented recommender system analyzes the frequency of medical events in the EHR and delivers a recommendation based on the preferred events. Therefore, our system works like collaborative filtering, i.e. items are chosen based on the patients’ rating history. This implies that our system, unlike systems implemented by Amazon, does not *use details of the registered user's profile (i.e., the physician).*^1^

The workflow of the implemented recommendation system requires the diagnose encoded b y International Classification of Diseases (ICDs), age, sex to predict the more probable Therapy Keys (TKs) per patient as parameters; and the following steps ensure:

- Synthesize the patient’s information and store the result with the relevant patient parameters: age, sex, Patient Identifications (ID), ICDs.
- Cluster ICDs and TKs. This step is required to reduce the dimensionality of both parameters (high number of items) and perform predictions of TKs group number depending on patient parameters, including ICDs groups.
- Train (deep learning model), validate, and export model to medical/hospital documentation and information system.
- Introduce a user interface for the recommender system, using the trained deep learning model based on the medical information system's data to recommend the treatment keys. As part of the combination of medical/hospital documentation and information/recommender system, this is the process where the physician accepts or discards the recommended TK in his/her professional autonomy and charges it via the charging system.

**Fig. 1:**
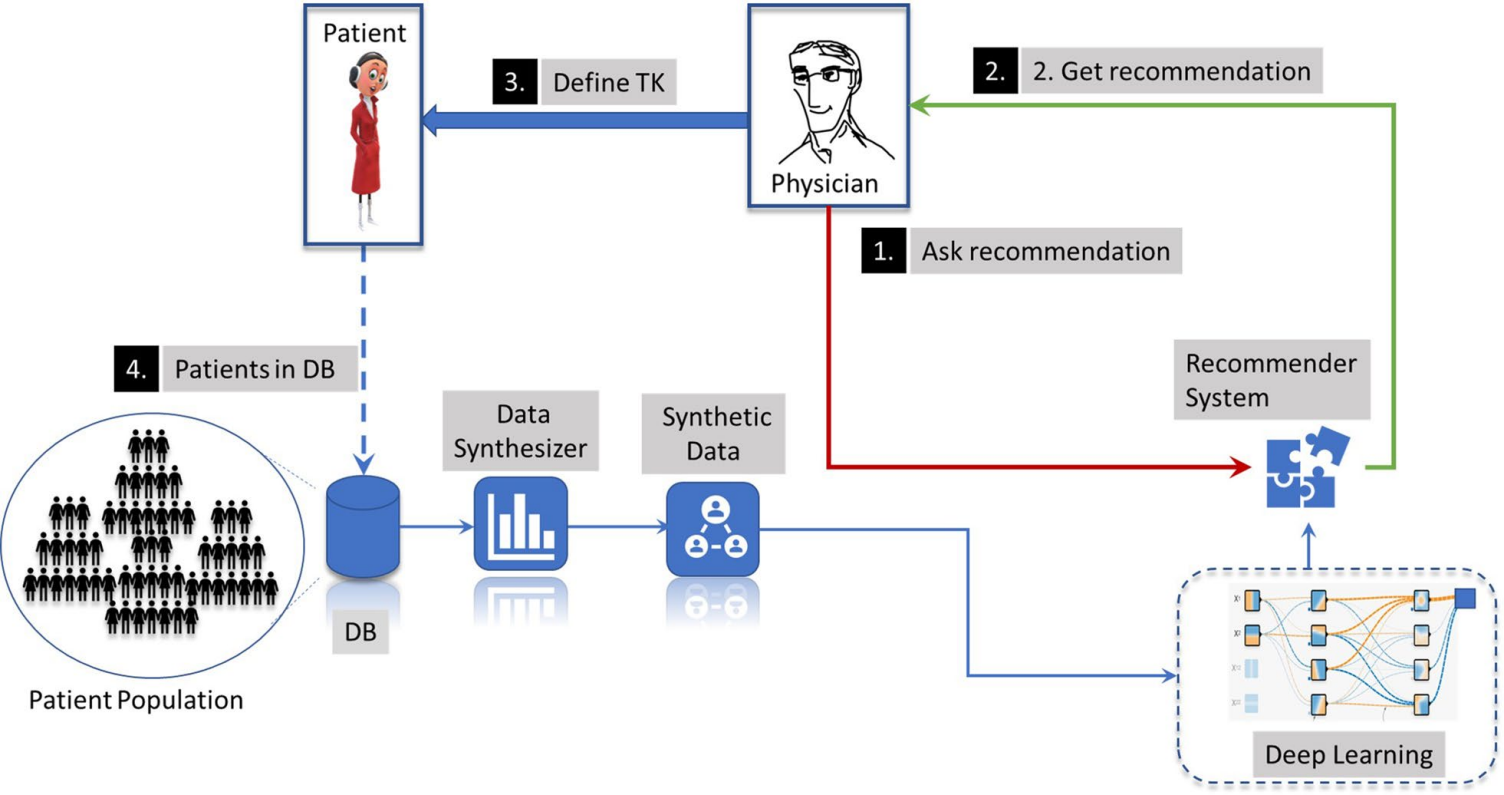
Structure of the implemented recommender system

The different steps in the workflow are resumed in Fig. 1. Once the recommender system is defined, using it implies the following steps:

1. The physician asks for a recommendation of the most frequently used TKs using the recommender system.
2. The physician gets a recommendation.
3. The physician selects the appropriate recommendations and does the treatment based on his own decision.
4. The new updated information about selected TKs is stored in the database (DB).

The implementation of this system is far from trivial, and we have to overcome several challenges:

- Data privacy: generating synthetic data from the original microdata containing confidential information so that they are safe to be released to users. Synthetic data are generated from sensitive records by replacing them with values simulated from probability distributions specified to preserve the actual observed data's key features.
- Data extraction: By using our techniques, we can synthesize large numbers of patients’ data, however, we also require robust techniques to filter large data amounts to synthesize data efficiently based on parallelization methods. To this end, data pre-processing was implemented using Spark.^2^
- Model selection: Selection of a robust model enables us to establish a relation between diagnoses and TKs.

In the following sections, we provide solution strategies for each one of these challenges.

### Modeling

#### Synthetic populations

Synthetic patients are generated from a representative patient database with samples of diabetic patients, some with heart insufficiency (Pakistan Database, from the UCI repository^3^). Diabetic patients with heart insufficiency often impose onerous requirements and restrictions for their lives. A clinical, as well as the economic impact, can be obtained with improved management of the disease based on a recommender system applied to Electronic Health Records (EHR) predicting “preference” (best clinical outcome) and “rating” (best reimbursement) that a physician would give to an item^4^ encoded in treatment keys (TK).

In the first instance, we only focus on heart insufficiency patients and combine this database with already known kinds of therapies. The goal is to combine the patient data with data from diabetic patients (group numbers extracted and analyzed in the recommendation system). To complement the data corresponding to the therapy, we would also include the treatment of heart insufficiency [13]. More than 90% of heart failure patients with reduced ejection fraction (systolic heart failure, or SHF, Group 1) and diabetes were treated with an ACE inhibitor (ACEi) or angiotensin receptor blocker (ARB) or with beta-blockers. By contrast, patients with diabetes and preserved ejection fraction (Heart Failure with Preserved Ejection Fraction, or HFNEF, Group 2) were less likely to receive these substance classes (*p* < 0.001) and had the worst blood pressure control (*p* < 0.001). Compared to patients without diabetes, the probability of receiving these therapies was increased in diabetic HFNEF patients (*p* < 0.001), but not in diabetic SHF patients. Aldosterone receptor blockers were given more often on diabetic patients with reduced ejection fraction (*p* < 0.001), and the presence and severity of diabetes decreased the probability to receive this substance class, irrespective of renal function.

Therefore, the hybrid database with typical TK and ICD groups (non-identifiable) combined with the Pakistan database for diabetic patients with heart insufficiency had the following attributes:

- Attributes from the Pakistan Database, including age and sex.
- Therapy groups depending on the kind of heart failure.

The total number of parameters, included input (V1 to V10) and output (O1 and O2) parameters, is illustrated in Table 1.

**Table 1:** Principal input and output parameters extracted from the HER of diabetic patients with heart insufficiency, according to the parameters contained in the original database

Input/output	Variable	Kind of parameter
–	ID	Character
V1	Sex	Binary
V2	Age	Real value
V3	Creatinine_phosphokinase	Real value
V4	Ejection_fraction	Real value
V5	High blood pressure	Binary
V6	Platelets	Real value
V7	Serum_creatinine	Real value
V8	Serum_sodium	Real value
V9	Smoking	Binary
V10	Anemia	Binary
O1	Time (feedback period)	Real value
O2	Treatment key TK (AEC, Aspirin or Beta Blocker – depending on heart failure group)	Binary

Notice that the parameter “Time” is the total number of days that the patient has been treated, in relation to the entire six months when this database has been obtained. Short times can mean that either the patient has recently entered into the system or that the therapy outcome was negative and/or the patient has decreased.

To better assess the quality of therapy, we used the treatment outcome as a reference parameter to generate a control population with a positive therapy outcome (no death patients).

**Fig. 2:**
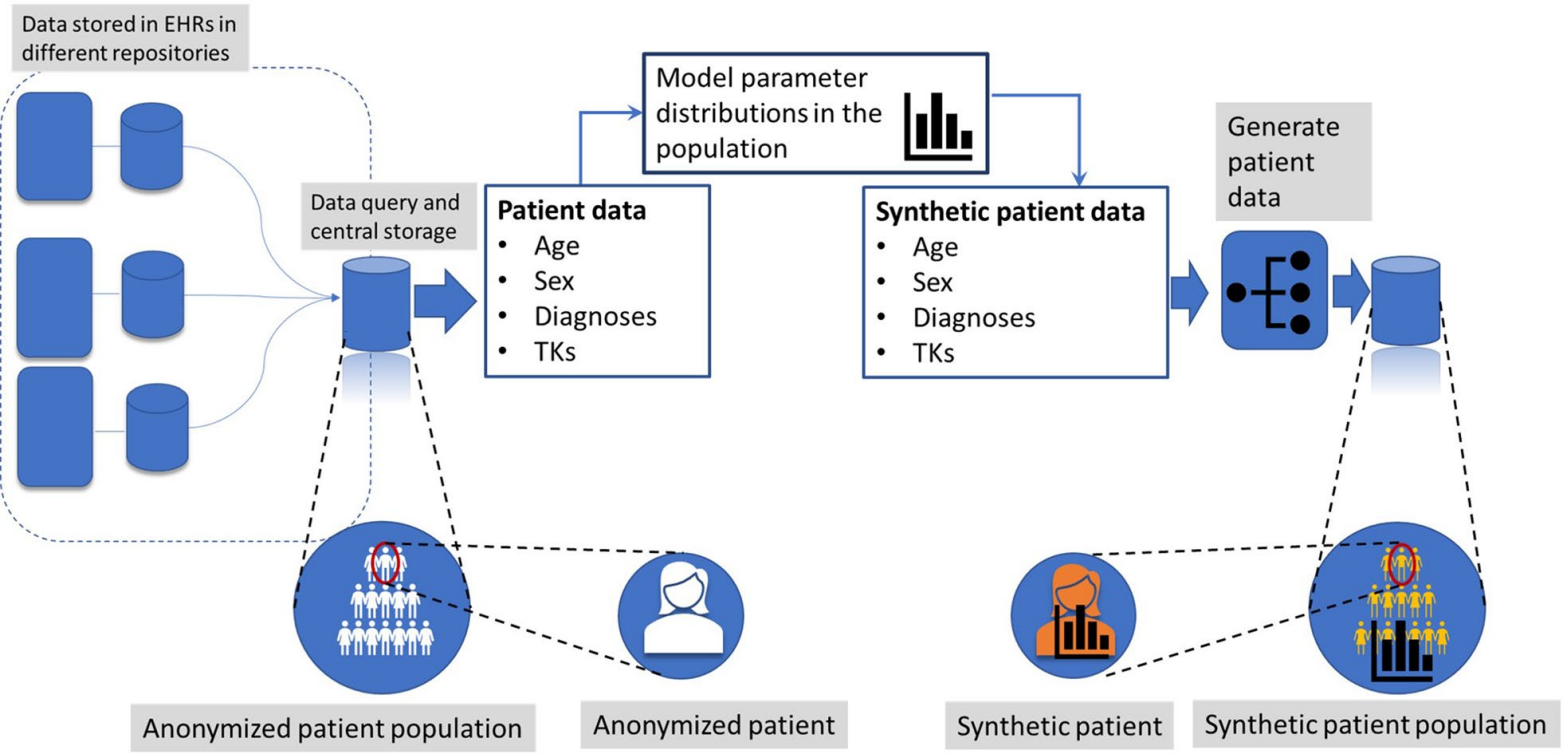
Workflow for the generation of synthetic patients. From this data, we analyze the parameter distribution and generate entirely new synthetic parameters using these distributions, which meant that we could generate synthetic patient populations completely and that this data provides more anonymization of the original clinical data

The steps required to generate the synthetic population are (see Fig. 2):

1. Querying of databases to extract a table with anonymized patient’s parameters; before modeling the patient’s clinical profile.
2. We model the distribution of each one of the parameters in the population and use the distribution functions to model and clone fully synthetic patients.
3. The data is stored in a database for further modeling when needed.

Personal information, like the identification number (ID), is randomly generated. For the simulation of the distribution of diagnoses and treatment keys (TKs, in Fig. 2), we used the “synthpop” package^5^ [14]*.*

The input data in our model is stored in csv files in a normalized format. Furthermore, since we are synthesizing data, there are no inconsistencies between the original and the synthesized data. To test the quality as well as the consistency of the synthesized data we estimated the mean distance of the frequency distributions $f\left( x \right)$ between the original ($x$) and synthesized ($\hat{x}$) variables (see Additional file 1: Table S1-1 in the supplementary section), an additional qualitative comparison between original and synthesized datasets for some parameters is presented in Additional file 1: Figure S1-1. A more detailed description of the performed data synthesis is provided in the supplementary material.

Observe that in this work we used public-available data, instead of data stored for instance in electronic health records. However, this architecture perfectly adapts to the analysis from real patient repositories. In such a case, each repository needs a Query engine that adapts easily into the Data definition of the Repository. Directly attached to the Query Engine is the “Normalizer” which outputs the data into the native format of the model. The normalized data provides the format in each line: Patient (Age, Sex, Laboratory Values) actual Diagnosis (encoded by ICD10), Date of Treatment, Treatment Key.

#### Deep-learning methods

The recommender system is based on the fuzzy classification system, such that the ICD groups, patients’ age, and sex are used as input parameters to predict the TKs.

To this end, a deep-learning method based on a multilayer neural net (NN) has been implemented. A dense NN is a type of artificial neural network (ANN) composed of multiple hidden layers, where every neuron in layer $i$ is fully connected to every other neuron in layer $i + 1$. Typically, these networks are limited to a few hidden layers, and the data flows only in one direction, unlike recurrent or undirected models.^6^

Extending the notion of a single layer ANN, each hidden unit computes $h_{i}$ a weighted sum of the outputs from the previous layer, followed by a non-linear activation $\sigma$ of the calculated sum as

1 $$h_{i} = \sigma \left( {\mathop \sum \limits_{{j = 1}}^{d} x_{j} w_{{ij}} + b_{i} } \right)$$

Here, $d$ is the number of units in the previous layer, $x_{j}$ is the output from the previous layer’s $jth$ node, and $w_{{ij}}$ and $b_{i}$ represent the weight and bias terms associated with each $x_{j}$.

Considering that neural networks emulate the spikes in neuronal processes, its result as a logical choice to select sigmoidal activation functions between different neurons in the different layers is clear. Recent investigations have demonstrated that rectified linear functions are the most effective in representing data processing in neural networks,^7^ particularly for networks with many layers, and thus more effective to process information [15]. In our investigation, we replaced these kinds of dense neuronal layers with continuous-valued logical operators in the last layers that can emulate fuzzy logical operations.

#### Continuous-valued logic multi-criteria decision operators and interpretability

Our strategy consists of implementing networks based on logical gates, modeled by Perceptron with fixed weights and biases. This hybrid neural model was introduced in [9, 10]. Here, a single Perceptron in the NN network is activated by so-called Squashing activation functions, differentiable, a parametric family of functions that satisfy natural invariance requirements and contain rectified linear units as a particular case [16, 17]. These Squashing functions approximate the cutting function in the nilpotent logical operators. A relevant characteristic of this family is its differentiability, which is vital for employing gradient-based optimization techniques.

In this investigation, we implemented the following function:

2 $$S_{\beta } \left( x \right) = \frac{1}{\beta }ln\left( {\frac{{1 + e^{{\beta \cdot x}} }}{{1 + e^{{\beta \cdot \left( {x - 1} \right)}} }}} \right),$$

where $\beta$ is a real nonzero value that needs to be adjusted to let the model be convergence.

Thus, the Perceptron in the neural networks' hidden layers can model a threshold-based nilpotent operator [9, 10]: a conjunction, a disjunction, or even an aggregative operator.

*This means that the weights of the first layer are to be learned, while the hidden layers of the pre-designed neural block, worked as logical operators with frozen weights and biases.* This means:

1. The first layer is trainable and has been implemented using an Exponential Linear Unit (ELU) activation function.
2. At the same time, the activation functions in the hidden layers, model the cutting function to avoid the vanishing gradient problem with the so-called squashing function $S_{{\beta _{{int}} }} \left( x \right)$ in the nilpotent logical operators (defined by Eq. 2), representing the internal layers. Besides logical operators, preference operators can also be modeled this way [10].
3. The final layer is again trainable, with a sigmoid activation function.

**Fig. 3:**
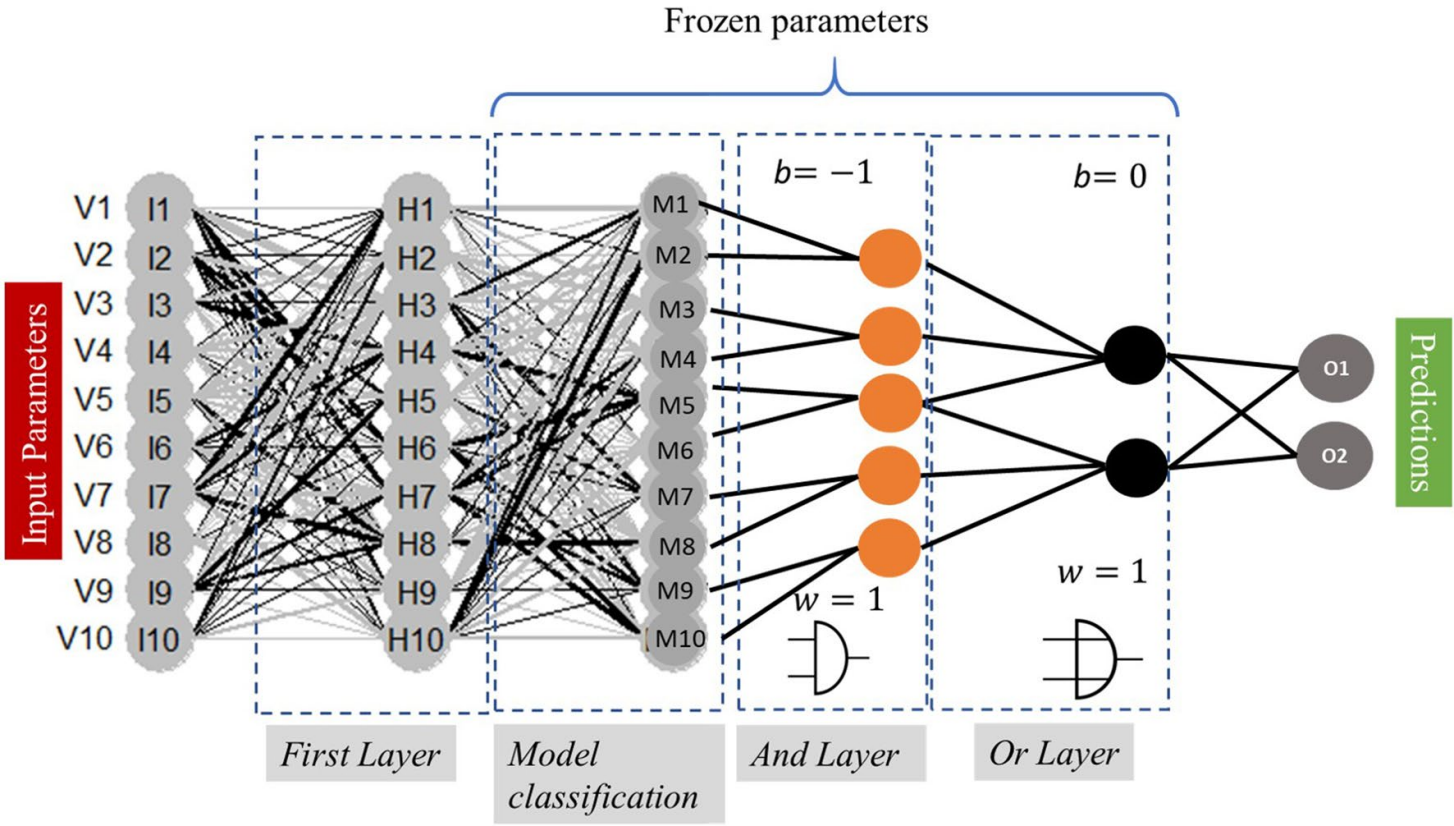
For now, with simplicity in mind, we implement two AND layers (conjunctions), followed by an OR layer (disjunction), to logically evaluate the nodes $M_{i}$, which is a process modeling human reasoning in the decision process

The weights in the first, $H_{i}$, and last layer, $O_{i}$, are optimized during training to establish an association between input $x$ and output y. In the second layer, we define the nodes $M_{i}$, layers with different and frozen weights $w_{{ij}}$ and biases $b_{{ij}}$ (see Eq. 1), grouping different relations between the input parameters. Thus, each of these nodes is essentially a hypothesis grouping of all the parameters with different statistical weights. Finally, the additional internal layers perform logical operations; some of them are resumed in Table 2. In Fig. 3, we illustrate this architecture, considering 4 $M_{i}$ nodes.

**Table 2:** Some examples of logical operators and their corresponding implementation (Csiszár et al. 2020c)

Logical operation	*w*_*ij*_	*b*_*i*_
AND	1	− 1
OR	1	0
NOT (x)	0	1
NOT (y)	− 1	1
Not (x) and Not (x)	− 1	1

In both the reference (Dense layers with “ReLU” activation functions) and our implemented Logic-Operator neural network (LONN) models, we implemented as a loss function the mean squared error ($MSE = \mathop \sum \limits_{{i = 1}}^{n} \left( {Y_{i} - \hat{Y}_{i} } \right)^{2}$) between labels $Y_{i}$ and predictions $\hat{Y}_{i}$. For the optimization process, we implemented an ADAM method, which is an algorithm for gradient-based optimization of stochastic objective functions [19], with a learning rate adjusted to 0.02 (see Table 4).

Due to that the categorical parameters were binary (for instance sex, smoke, anemia), we converted them to 1 and 0 s. The other numerical parameters were normalized using the normalization function $norm\left( x \right) = \left( {x - min\left( X \right)} \right)/\left( {\left( X \right)~ - min\left( X \right)} \right)$, where $min\left( x \right)$ and $max\left( x \right)$ are the minimal and maximal values of the vector $X$ where $x$ belongs. With these data transformations, we were able to get a homogeneous input matrix for the deep-learning model.

Finally, we selected 80% of shuffled data for training to get a balanced sample for training and avoid eventual biases as well as overfitting in the training process. The list of the optimizer hyperparameters are listed in Table 3, while the list of model hyperparameters are listed in the Table 4. These parameters were manually tuned. An implementation of automatically tuned parameters will be presented in a future work.

**Table 3:** Hyperparameters of the ADAM optimizer for a dense Layer network ReLu and LONN

	Tested range	Selected value
batchsize	32, 50, 100	50
lr (learning rate)	0.01, 0.02, 0.05	0.02
epochs	50, 100, 500	100

**Table 4:** Model-specific hyperparameters for comparison a dense Layer network ReLu vs. a LONN

	NN–dense layers/ReLU activation	LONN
$\beta _{{int}}$	–	1.5
Learning rate (lr)	0.02	0.02
# Internal layers	4	4
# Neurons per layer	10, 4, 3, 2	10, 4, 3, 2
# Trainable parameters	293	116
# Non-trainable parameters	0	59

**Fig. 4:**
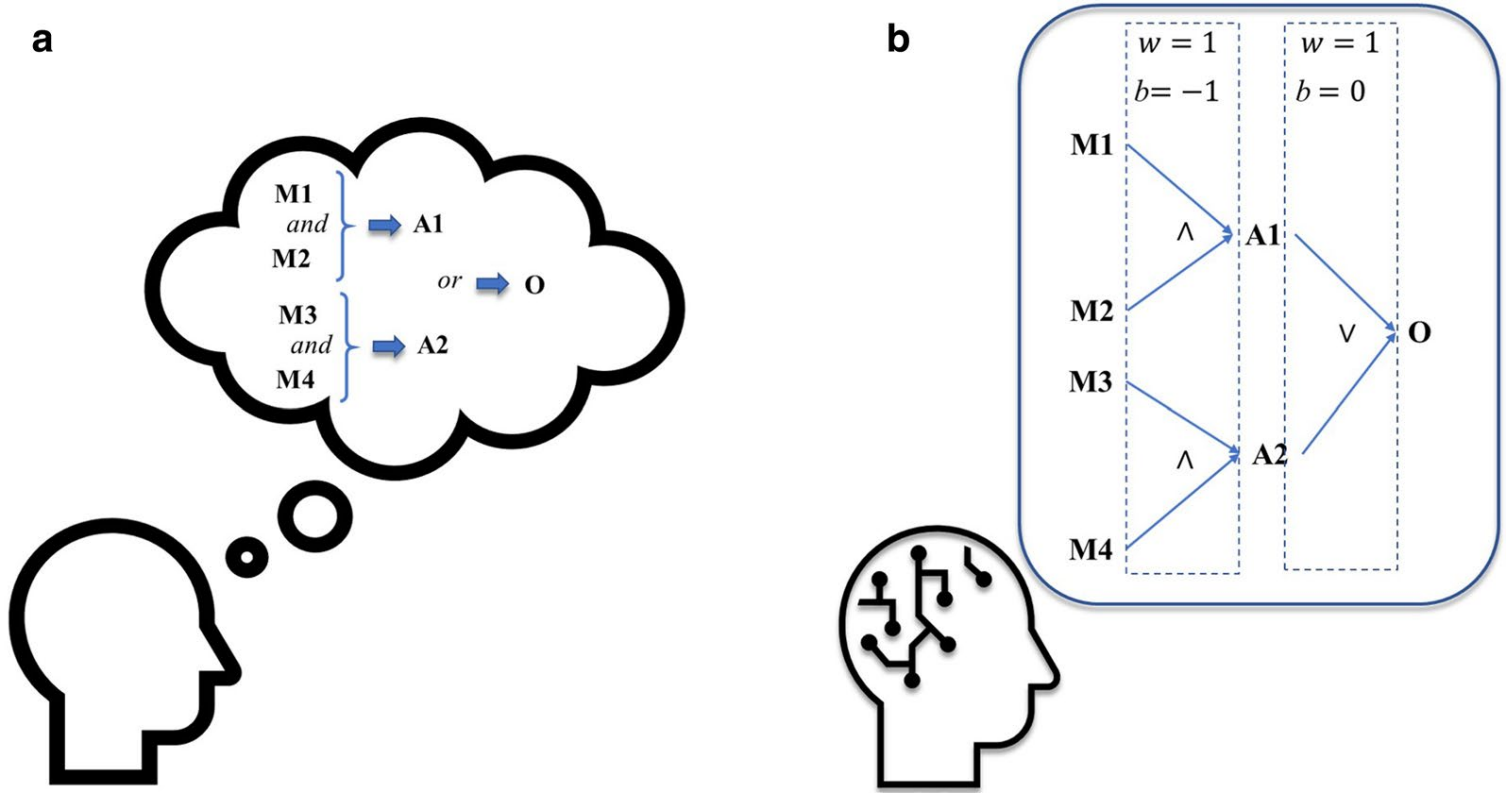
Representation of natural thinking processes (**A**) and by neural networks (**B**). Logical processes, like the combination of “and” and “or” processes, are implemented as fuzzy logic, representing natural uncertainties in the thinking process

Therefore, our LONN model simulates cognitive processes like rational, logical thinking process, considering that this logic is joined by fuzziness, i.e., logical operations are not exact but essentially fuzzy due to the implemented continuous-valued operators (see Fig. 4) [20].

Even though we were inspired in this design by cognitive processes, our aim is not to reproduce the native human thinking process in silico but rather to implement processes much closer to the natural processes in making information processing interpretable. Furthermore, we aim to retain a flexible modeling method that can be applied in different and relevant medical environments: the model needs only a few parameters out of the medical space, but addresses the major medical workflow, from medical analyses to diagnoses and treatment selection. The main relevant result is plausible suggestions of medical treatments. That should be applicable in nearly all medical domains.

## Results

### Model validation

The ANN has been implemented using tensor flow in an R environment. The data ingestion and pre-processing are made with Spark. The data ingested in the model is normalized, and the internal model validation is performed using this normalized data. From this data, we use 80% for model training and 20% for model validation.

We implemented a control model using “ReLU" activation functions with the same topology as the LONN. The main model hyperparameters of both the control and the continuous-valued multi-criteria network are listed below in Table 4. Observe that we fixed the model architecture, i.e. the number of layers and neurons was constant for all the tests.

**Fig. 5:**
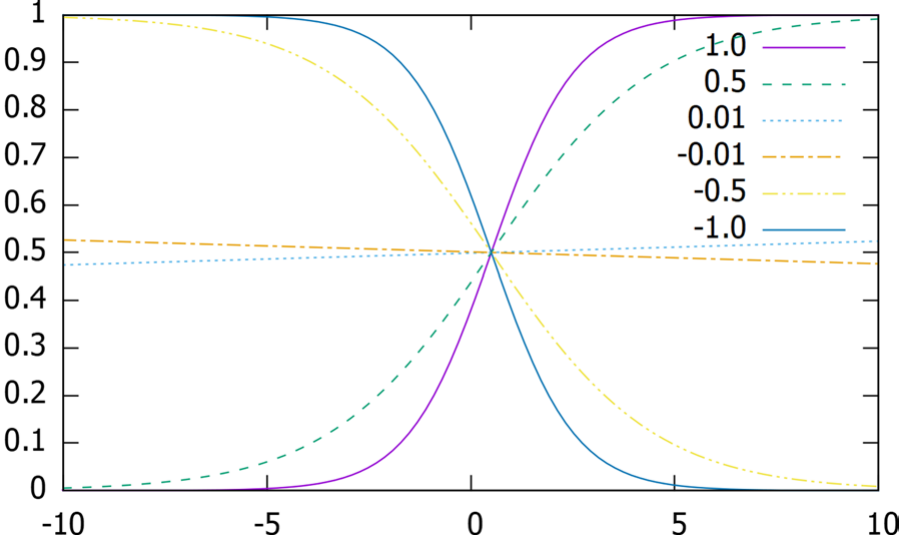
Squashing function $S_{\beta } \left( x \right)$ for different parameters $\beta$

For the parameters $\beta$, we performed different validation runs to explore the optimal value for the first ($\beta _{{inp}}$) and internal ($\beta _{{int}}$) layers. The effect of this parameter on the slope of the function $S_{\beta } \left( x \right)$ is shown in Fig. 5.

Observe that the Squashing functions go through the point (0.5, 0.5) for all values of $\beta$ and that:

$$\begin{aligned} \beta & > 0,~squashing~function~is~increasing; \\ \beta & < 0,~squashing~function~is~decreasing. \\ \end{aligned}$$

Moreover, $S_{\beta } \left( x \right) = S_{{ - \beta }} \left( {1 - x} \right)$ holds, i.e., $S_{{ - \beta }} \left( x \right)$ is the reflection of $S_{\beta } \left( x \right)$ over the axis x = 0.5. This means that in the interpretation, for a negative $\beta$ value, a negation operator is applied.

**Fig. 6:**
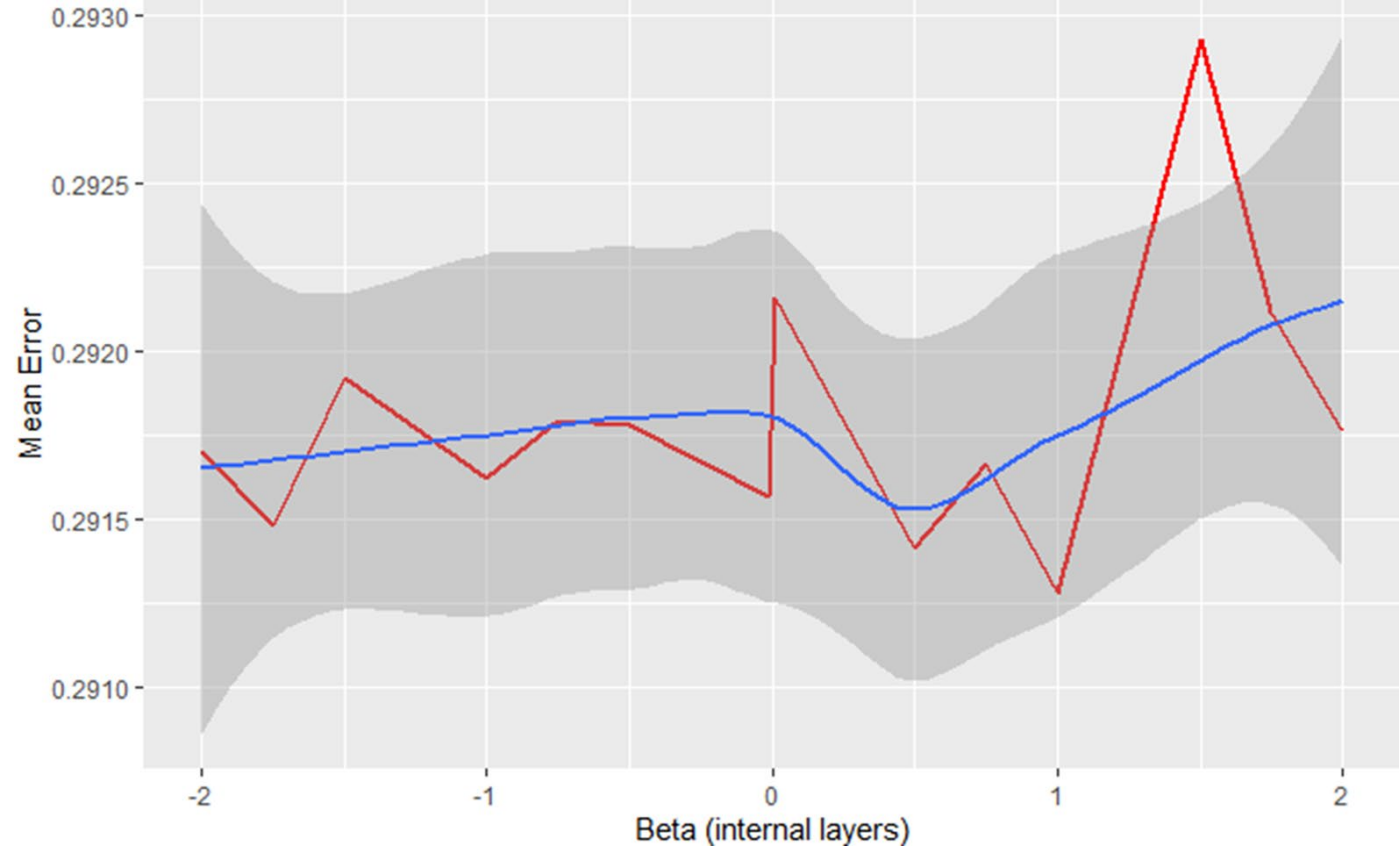
Training error of the model presented in Fig. 3 for different $\beta _{{\text{int} }}$ (x-axis) estimated after 50 epochs for each validation run

The systematic test of the mean error considering different $\beta _{{int}}$ (Fig. 6) implies that the relative fluctuation of the error is larger for $\beta > 0$. But the variations are extremely minimal and have no significant influence on the final training error.

**Fig. 7:**
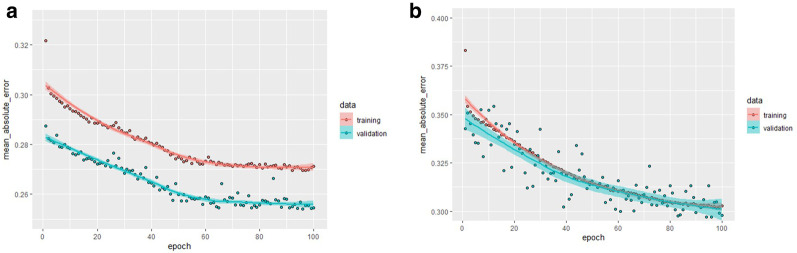
Internal model validation with a dense NN (two internal layers, with 10 and 4 neurons with ReLU activation function, **A**), versus the logic-operators NN (LONN, **B**) for 100 epochs

We performed a model validation with these parameters in mind and compared the dense control layer NN and the LONN shown in Fig. 3. The results are presented in Fig. 7.

At first glance, we observe that the model defined with continuous-valued logic multi-criteria decision operators is less accurate (training error 29.79%) than conventional networks (training error 25.46%).

**Fig. 8:**
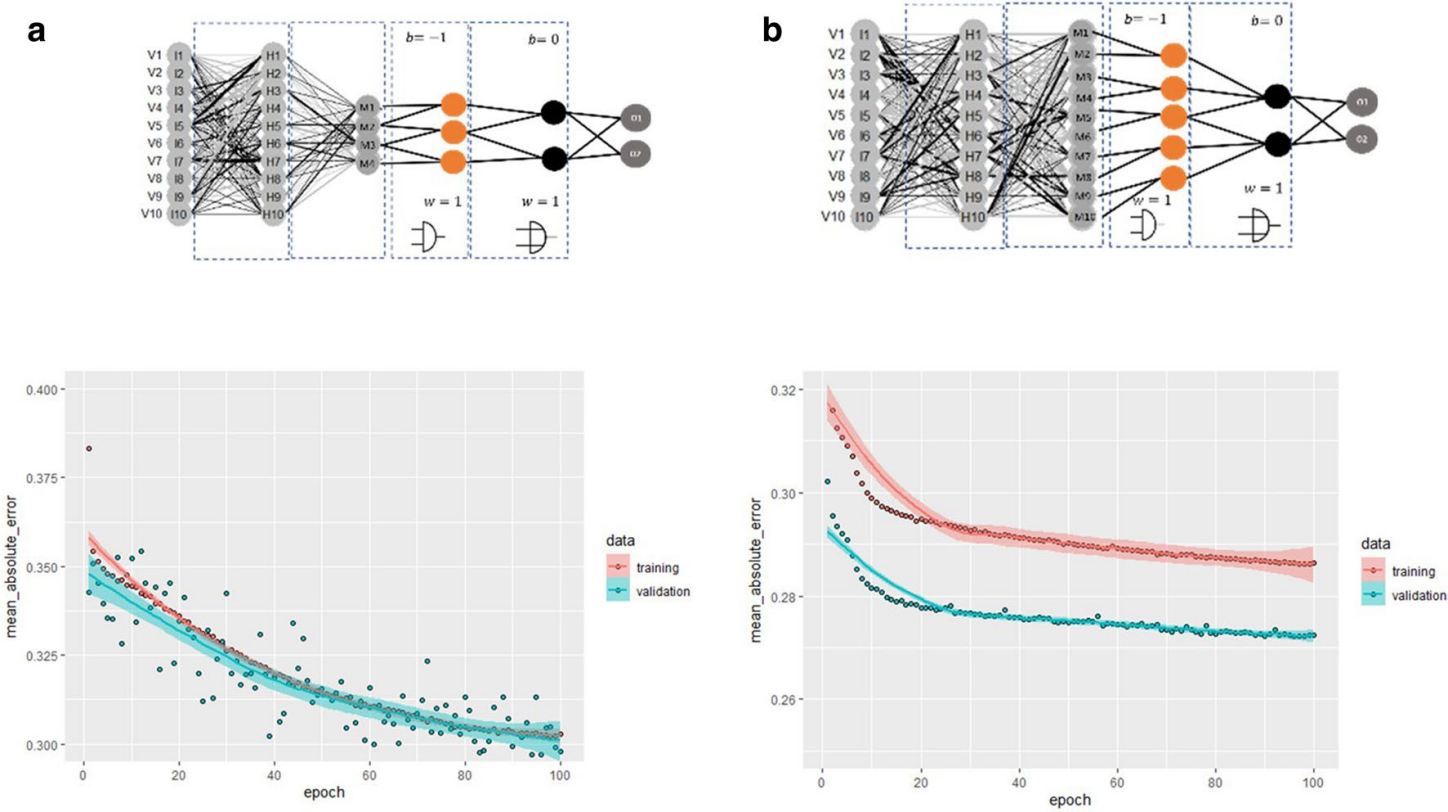
Test of two LONNs with two different topologies with different numbers of internal layers and logical operators

An inspection of the dependency of the model accuracy on the number of neurons in the internal layers (4 $M_{i}$ neurons, Fig. 8A, and 10$M_{i}$ neurons with a corresponding modification of the connection of the logical operators, Fig. 8B) demonstrates that an increase of the number of neurons has a slight influence on the mean absolute error (training error 27.24%), i.e. as expected, the increase of the number of neurons in each layer minimizes the training error.

We finally tested the overall accuracy of the model against test data by computing the frequency of true positives for the predicted therapies as well as the mean squared error of the predicted therapy time (see Table 5). The overall accuracy of the model could be improved by increasing the number of neurons per layer as well as the number of layers, but the training error was higher than the validation error (Fig. 8B). For this reason, we conclude that the quality of the model 8A is higher; for this model, we additionally found a precision of 64.14% and a recall of 98.43%. The low accuracy and precision of the model are perhaps originated in the available database used for this model; we expect to obtain more accurate results when these models will be trained on data from real EHRs.

**Table 5:** Measured accuracy of LONN models from Fig. 8

	LONN-network A (%)	LONN-network B (%)
Training error	29.79	27.24
Accuracy—therapy prediction	63.49	64.00
RMS error—therapy time	32.15	33.02

In a nutshell, the observed loss of accuracy in fuzzy logic networks is compensated by the model interpretability and by the fact that we dramatically reduce the number of trainable parameters.

### Model consumption and visualization of recommendations

**Fig. 9:**
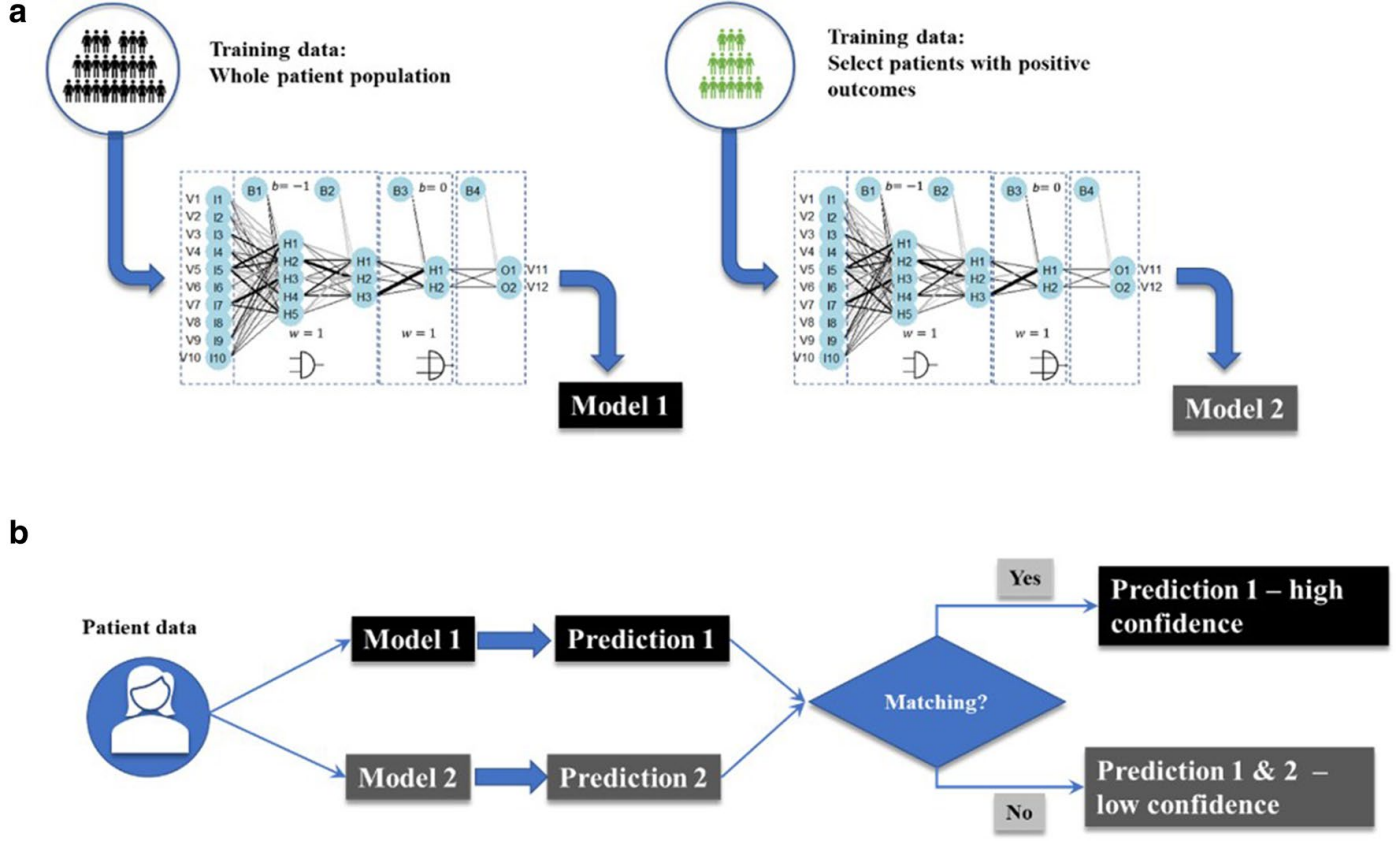
Training method for the recommender system. We train two different models based on the whole data set (Model 1) and a dataset consisting only of positive outcomes (Model 2). After that, we use both models to make different predictions. If there is a matching in the predictions, then Prediction 1 is used as the standard with high confidence; otherwise, predictions from Model 2 are provided but have low confidence

We have trained two different models for the final model consumption, one with the whole database (Model 1, Fig. 9A) and another with a database that selects only positive outcomes (Model 2, Fig. 9A). The goal of this training method is to make predictions based on the positive outcomes and then evaluate the confidence of the prediction:

- High confidence when the predictions of both models overlap (Fig. 9B)
- Low confidence when there is no matching. In this case, we provide recommendations based on the outcomes of model 2, but with a warning that this prediction has low confidence (Fig. 9B)

In this way, we aim to set up a recommender system, leading to outcomes that should be almost positive for the patients.

The trained model is finally exported and used for consumption and data ingestion (as shown in Fig. 1):

- The physician asks for a recommendation once he has a clear diagnosis of the patient, correspondingly encoded in ICDs.
- The trained model ingests the ICDs, as well as the sex and age of the patient, and delivers corresponding TKs recommendations encoded as group numbers.
- The final TKs are decoded.
- The final recommendation is finally deployed and visualized, for instance, in an application or the physician’s software.

To improve the model interpretability, we require a visualization of the distribution of the input parameters, automatically generated by the model, as shown in the supplementary Sect. 2 (Additional file 1: Figure S2-1). From this result, we discover a parameter hierarchy, with the creatinine concentration (serum and phosphokinase) as the relevant parameter. This result makes sense regarding the fact that the creatinine concentration is a metabolite that indicates how good the patient's adherence to therapy is [21].

**Fig. 10:**
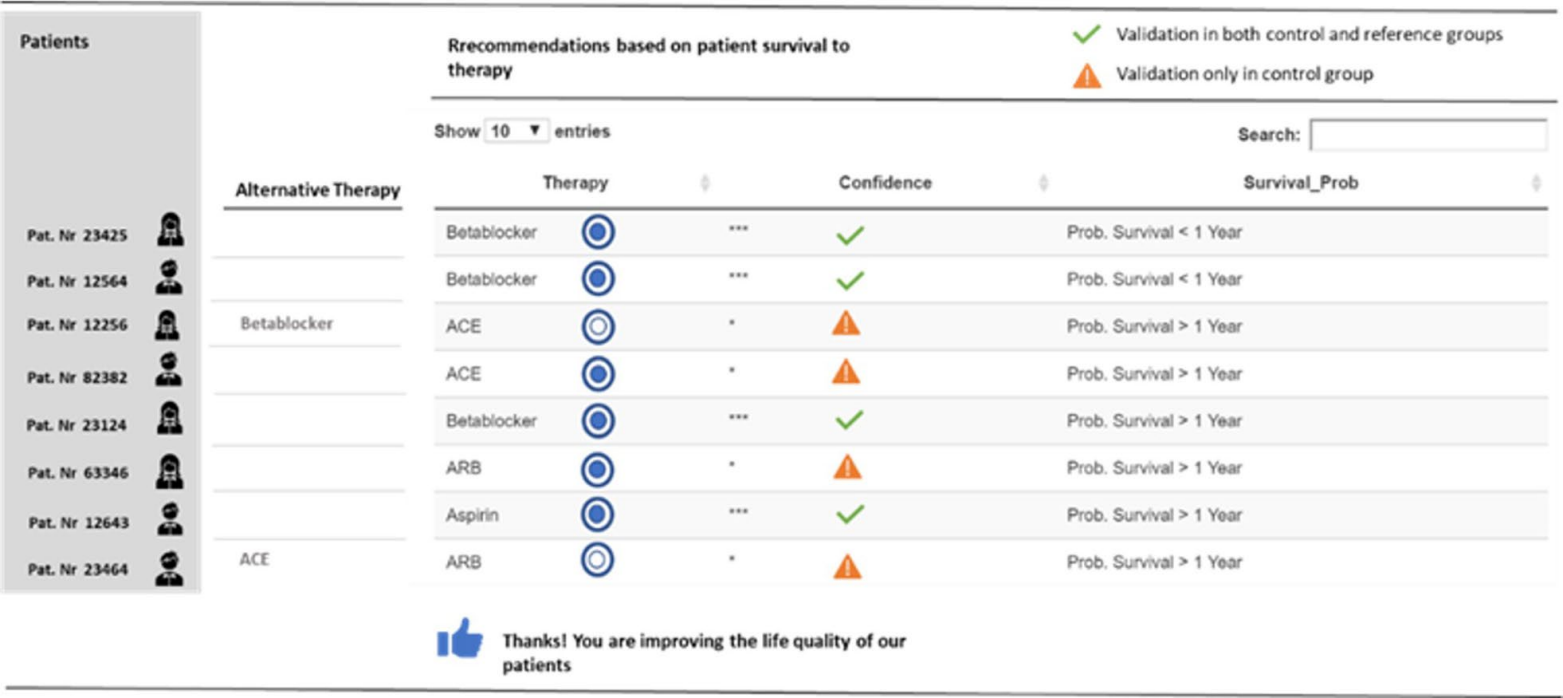
Diagram of a potential app based on the model outcomes

The final recommendations are then visualized in a dashboard, as is shown in Fig. 10. Not only the recommendations but also the confidence of the prediction based on positive outcomes (binary value: 1 for confidence, 0 for non-confidence) is visualized. In the case of low confidence, the two alternative treatments from the two trained models are deployed. In this Dashboard, the physician can provide subjective feedback about how useful or accurate are the deployed recommendations.

Finally, indifferent to whether the recommended TKs are accepted or not by the physician, the recommendation is stored in the database. This final step implies that the database is continuously updated with new information, implying that models have to be trained periodically to guarantee their quality. This implies that a robust implementation of this system requires an automatic system that allows its new training.

## Discussion

As it is well-known, deep learning methods are advantageous because they allow the modeling of non-linear systems while at the same time being robust against small changes of training data, unlike methods as random forest.^8^ With our implemented methodology, we aim to further improve deep learning methods in the following three ways:

- *Interpretability* Implementing a combination of logical operators/gates, i.e., the different parameters are logically combined. Since the parameters of the deep layers are frozen, we can rely on the parameter classification in the first layer, which provides interpretable information about how a parameter hierarchy influences the model outcome. Furthermore, these operators are implemented using fuzzy logic, which is closer to natural thinking processes.
- *Safety* The statistical weights and biases in the internal layers are frozen, i.e., they do not change in the training process. This guarantees that these parameters are robust, even when adversarial examples are employed.
- *Increased efficiency* Fewer parameters to be trained.

These aspects are extremely relevant when neural networks are implemented in a medical field, not only because of the high safety standards required but also because physicians need to understand how the network delivers results. With our implementation of the LONN in a deep-learning environment, we have demonstrated that we can implement more transparent models with highly efficient computational tools.

Observe that in this approach we are providing a rather precise interpretation for an epistemological problem in machine learning that has not been fully solved [22]: with our implemented LONN we know that from the extracted features in the layer $M_{i}$, we perform a logical combination of the recommendations, i.e. we know which are the internal operations in the network. However, our LONN implementation is partially interpretable since the feature extraction has been trained in the Keras environment and cannot be fully interpreted. For this reason, with our methodology, we can better justify a result, and we can provide a partial explanation about how the algorithm works, without pretending to provide a full causal relation between the input parameters and the model outputs.

Several problems will persist in the implementation of recommender systems in medicine. Patient databases are dynamic and evolve depending on the disease distribution in the patient population, which can eventually invalidate trained models. Once activated, recommender models can influence physicians' decision-making, which will be reflected in how treatments are suggested to patients who are registered in the database. This fact implies a co-evolution of the database and the implemented AI model coupled to the recommender system, considering that AI models need to be trained and validated regularly.

Besides selecting the correct methodology to process and analyze the data for predictive modeling, the most challenging problem is protecting patient’s data. When it comes to data protection, three main aspects are problematic: The access to the patient’s data for machine learning is limited as it always requires the patient’s consent; recommendation systems can generate incorrect patient profiling, which simultaneously can be misused; Physicians seldom get enough support in their day-to-day work.

In the real world, the data is protected in a way that you may not get enough data to train the network sufficiently. To solve this contradiction the idea of synthesizing came to attention, to produce larger amounts of training data with the same properties of the real data, but with less demand of repetitive access to the real data. This problem is solved by generating databases with synthetic patients. In solving this problem, we used a small random but representative dataset of anonymized representative patients to model their individual clinical data with characteristic distribution patterns to generate completely synthetic patients. We have also developed our methodology and workflow, based solely on this synthetic database. Naturally, after training models on synthetic data, models can be further trained on real patient data with consent inside closed repositories.

The other two aspects are critical since it concerns the rights that patients do not have to be subject to decisions based solely on automated processing [23]. For this purpose, patients reserve the right to be informed about the existence of automated decision-making, including profiling, and their right to receive meaningful information about the logic involved and the significance and the envisaged consequences of such processing. However, whatever is recommended to a physician, it needs to be seen as a recommendation. The final decision on how a patient receives treatment will always be the physician’s call.

The behavioral features involved in the decision-making process, i.e. how a recommender system may influence physician’s decision making [24], and how this technology could have a positive impact on patient’s care and better economic management is a problem that not only depends on the technical implementation and the AI component but also on the final user’s interface. For instance, in a recent investigation, it has been shown that*, when designed well, recommender systems that incorporate treatment costs can result in significant cost savings, while providing similar or better health outcomes. Scenarios, where practitioners do not feel time pressure and have access to accurate cost information, are most conducive for adopting recommendations and creating change* [3]*.*

A deeper analysis of this aspect lies beyond the scope of the present POC implementation and has to be analyzed in the future once the recommender system and its corresponding user interface run in real conditions.^9^

## Conclusion

We have implemented a recommender system based only on the statistical analysis of data stored in HER and working as collaborative filtering. The implemented system estimates the therapy time and treatment keys, TKs (in this case the use of ACE, Aspirin, or Beta Blocker), and implements deep learning to predict TKs depending on the patient's diagnoses and essential phenotypic information.

We can demonstrate that our methodology reaches a training accuracy of 72%. This accuracy is lower than the one obtained using conventional NN implemented with dense layers and ReLU activation functions and more dense layers. However, in this kind of implementation, the interpretability (architecture and emulation of rational decision processes) and safety (parameters of internal layers are frozen) are two characteristics that are perhaps much more valuable than the accuracy of the model.

Observe that we are reporting a proof of concept for the application of this algorithm for recommender systems and that the current report is based on publicly available data that has a lower quality than the data stored on real EHRs. In planned application and trial on an electronic health record (EHR), we will first re-evaluate the accuracy and precision of the algorithm and then compare the outcome of the model with the outcome of Physicians without using the Model. We expect a similar accuracy and precision between the model and the physician’s performance.

In the next step plan to use this methodology with data from electronic health records. This first Trial will require special techniques to query and ingest large amounts of data. Furthermore, this implementation will allow us to implement a feedback loop including not only new data to retrain our models, but also feedbacks from customers informing whether they accept the recommendations and if they are satisfied with the system. This last information is relevant for an eventual better categorization of the input parameters. Thus, it is relevant to point out that the recommender system coevolves with the whole system, i.e. the recommender system can eventually influence physicians' decisions, while such decisions, stored in the databases, can influence the recommender system when it is periodically re-trained. Such coupled dynamics between model re-training and model used by physicians is fundamental since such models are not static and dependent on how they are used. Such evolution has to be the focus of future analysis, considering behavioral aspects related to the use of recommender systems by physicians.

Finally, recent advances in neural networks allow the visualization and recording of the learning process to leverage the safety and transparency in the use of deep learning methods [25]. Such methods have to be implemented in the future to increase the safety in the validation and use of these methods in the medical field.

## Supplementary Information

**Additional file 1**. Supplementary tables and figures.

## Data Availability

All models and relevant data inputs are available upon request to the corresponding author.

## Abbreviations

TK: Therapy key
EHR: Electronic health record
SVM: Support vector machine
AI: Artificial intelligence
DB: Data bank
SHF: Systolic heart failure
HFPEF: Heart failure with preserved ejection fraction
ID: Identification number
ANN: Artificial neural network
NN: Neural network
LONN: Logic operator neural network
ICD: International classification of diseases

## Footnotes

1 https://towardsdatascience.com/introduction-to-recommender-systems-6c66cf15ada

2 https://spark.rstudio.com/

3 The data has been obtained from the [uci repository] (https://archive.ics.uci.edu/ml/datasets/Heart+failure+clinical+records).

4 In this case, the best treatment.

5 https://www.r-bloggers.com/generating-synthetic-data-sets-with-synthpop-in-r/

6 https://en.wikipedia.org/wiki/Multilayer_perceptron

7 https://scholarworks.utep.edu/cgi/viewcontent.cgi?article=2170&context=cs_techrep

8 https://christophm.github.io/interpretable-ml-book/other-interpretable.html

9 All products which offer decision support must be built under the design and software development process for medical devices.
