# Supplementary material for: Medical recommender systems based on continuous-valued logic and multi-criteria decision operators, using interpretable neural networks
Source: BMC Med Inform Decis Mak. 2021 Jun 11;21:186. doi: 10.1186/s12911-021-01553-3 (PMC8194023; doi:10.1186/s12911-021-01553-3)
Supplement: Supplementary file 1 — Additional file 1. Supplementary tables and figures. [file 12911_2021_1553_MOESM1_ESM.docx]

# Supplementary 1

For the simulation of the distribution of diagnoses and treatment keys (TKs, in Figure 2), we used the “synthpop” package^[[1]](#footnote-1)^ (Nowok et al., 2016). *Synthesis is performed on a variable-by-variable basis by ﬁtting a sequence of regression models and drawing synthetic values from the corresponding predictive distributions. The ﬁtted models are conditioned on the original variables so that the number of covariates increases for subsequent variables. In this approach, models can be deﬁned for each variable separately, and structural features of the data, such as logical constraints or missing data patterns, can be considered.*

In this research, we implemented linear regression and norm rank^[[2]](#footnote-2)^. Using this method, synthetic values of normal deviates of ranks of the values in $y$ are generated using the spread around the fitted linear regression line of normal deviates of ranks given $x$. Then normal synthetic deviates of ranks are transformed back to get synthetic ranks, which are used to assign values from $y$. First, the regression coefficients are drawn from a normal distribution with mean and variance from the fitted model for proper synthesis.

The regression is carried out on Normal deviates of ranks in the original variable. Synthetic values are assigned from the original values based on the synthesized ranks that are transformed from their normal synthesized deviates. With the applied method, we obtained an error^[[3]](#footnote-3)^ of about 1% for all the relevant parameters (see Table S1-1 as well as Figure S1-1)

*Table S1-1Error of the synthesized parameters*

| **Variable** | $\Delta\underline{f}$ |
| --- | --- |
| Sex | $0$ |
| Age | $1.31\times{10}^{-16}$ |
| Creatinine_phosphokinase | $4.16\times{10}^{-17}$ |
| Ejection_fraction | $1.24\times{10}^{-16}$ |
| High blood pressure | $0$ |
| Platelets | $-3.0\times{10}^{-16}$ |
| Serum_creatinine | $-5.12\times{10}^{-17}$ |
| Serum_sodium | $-7.18\times{10}^{-17}$ |
| Smoking | $0$ |
| Diagnose (kind of heart insufficiency - HF) | $0$ |
| Time (feedback period) | $1.58\times{10}^{-17}$ |
| Treatment key TK (AEC, Aspirin or Betablocker) | $-4.44\times{10}^{-16}$ |

A qualitative representation of the synthesized parameters is presented in [**Figure S1**](#bookmark=id.2jxsxqh)**-1.**

**
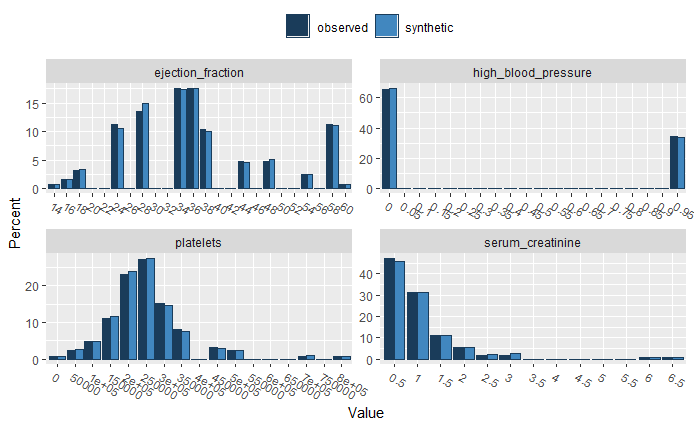
**

*Figure S1-1 Boxplot of error distributions of different exemplary parameters in the dataset*

To generate a complete synthetic population, we modeled and simultaneously synthesized the data $N$ for new patients, such that the final population is the number of the initial patient population multiplied by the number of clones per synthesized patient. We performed an arbitrary synthetizing of 10 patients for each patient, implying that we generated a synthetic population of approximately $N=2000$ individuals. In this way, we obtained an extended database to apply deep learning and have a large population with balanced characteristics, but we restricted the maximal number of clones to avoid overfitting the models trained on this database.

Once the database is defined, we introduced efficient methods for data filtering based on Spark, for cluster-computing with implicit data parallelism^[[4]](#footnote-4)^.

# Supplementary 2

We use the weight distributions $w_{ij}$ from equation 1 to assess the parameter classification in the different network layers.

The weights between the input parameters $V_{i}$ and the first layer $H_{i}$ are the single parameters (besides the output layer) that can be trained in this model; in Figure S2-1 we present the values obtained after 100 epochs as a heat map, where the x-axis represents the mean weight of the link of each of the input parameter (from V1 toV10) on the first trainable layer (from H1 to H10; see model architecture in Figure 3). In the same heatmap we also deploy the corresponding annotations of the input variables and first layer neurons as a parameter clustering computed using k-means.


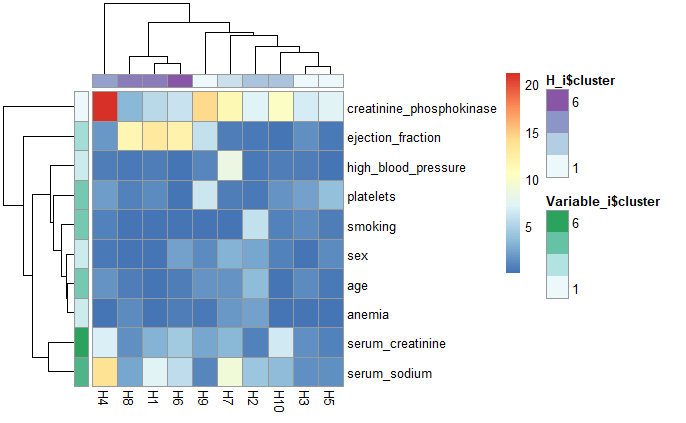


*Figure S2-1 classification of the model weights* $w_{ij}$ *in the first trainable input layer (H1 to H10) with the annotations of the first layer neurons (H_i) and input variables (Variable_i) after 100 epochs*

This information is useful to establish the hierarchy of the input parameters in the NN model and presents a clear interpretation as to which parameters are more relevant for the trained model: both parameters creatinine Phosphokinase and serum creatinine are particularly relevant for the final prediction of therapy outcome and therapy duration. Serum Creatinine Test and Creatinine Phosphokinase are waste products from the normal breakdown of muscle tissue. As creatinine is produced, it’s filtered through the kidneys and excreted in urine and is an indicator of the normal function of the kidney as well as its capacity to expel metabolites generated during a patient’s treatment. Therefore, the current result suggests that the metabolization of the substances provided in therapy is relevant for the estimation of the success of the therapy outcome. Furthermore, the observation of the creatinine is an important indicator of how good the patient’s compliance and adherence to the therapy is, which is naturally directly correlated to the therapy’s outcome^[[5]](#footnote-5)^.

Therefore, the analysis of the parameter distribution in the first layer is from a medical perspective meaningful and can be used as a proxy for a health professional.

The analysis of the model classification in the next layer is less meaningful from a medical perspective since it is a random definition that remains frozen in the training process. Essentially, this layer groups relations between the classified input parameters $H_{i}$ differently and remains frozen during all the training process. This explains why there is no convergence, i.e it preserves its initial random configuration during the training process (Figure S2-2).


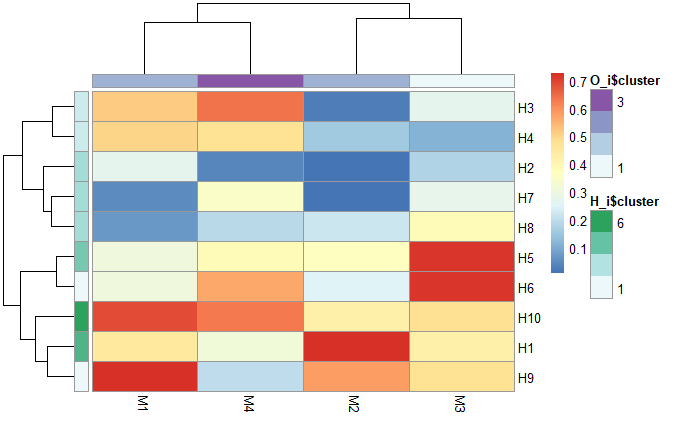


*Figure S2 -2 Distribution of model features* $M_{i}$ *encoded in the second -non-trainable- layer (from H1 to H10) in the LONN model (Figure 3), with the annotation of the H (H_i) and O (O_i) layer*

The further classification of $M_{i}$, which is basically a feature extraction based on the parameter hierarchy, as is shown in Figure S2-2, is then performed with the continuous logic multi-criteria operators (Figure 3). In this result we also computed the corresponding annotations using a k-means method. This means features extracted from the initial parameter hierarchy are evaluated using the continuous logic operators implemented with our LONN methodology.

1. <https://www.r-bloggers.com/generating-synthetic-data-sets-with-synthpop-in-r/> [↑](#footnote-ref-1)
2. <https://www.unece.org/fileadmin/DAM/stats/documents/ece/ces/ge.46/20150/Paper_24_bnowok_synthpop.pdf> [↑](#footnote-ref-2)
3. Difference between the number of observed and synthetic data in percent [↑](#footnote-ref-3)
4. <https://en.wikipedia.org/wiki/Apache_Spark> [↑](#footnote-ref-4)
5. <https://academic.oup.com/jat/article/40/8/659/2445890> [↑](#footnote-ref-5)
